# The Visualization of Intraneural Venous Compression in Trigeminal Neuralgia by Using Advanced Three-Dimensional Computer Graphics: An Illustrative Case Report

**DOI:** 10.7759/cureus.57935

**Published:** 2024-04-09

**Authors:** Seisaku Kanayama, Naoyuki Shono, Masato Inoue

**Affiliations:** 1 Neurosurgery, Center Hospital of National Center for Global Health and Medicine, Tokyo, JPN; 2 Neurosurgery, The University of Tokyo Hospital, Tokyo, JPN

**Keywords:** surgical planning, 3d-computer graphics model, offending vein, microvascular decompression, trigeminal neuralgia

## Abstract

Trigeminal neuralgia (TN) caused by venous compression presents challenges in surgical management, unlike the arterial type. Preoperative diagnostic certainty regarding venous etiology and anatomical relationships is crucial for surgical success. We discuss a case of TN caused by a vein passing through the nerve that was challenging to visualize on conventional MRI and was treated successfully by leveraging information from modern surgical simulation technology with 3D computer graphics. We recognized a potentially troublesome anatomical feature in advance and mitigated the risk by identifying a collateral drainage route for the causative vein, making it feasible to be sacrificed while ensuring treatment efficacy.

## Introduction

Trigeminal neuralgia (TN) is mainly caused by neurovascular compression (NVC). NVC typically results from arterial compression at the trigeminal root entry zone (REZ), and venous compression is less common (around 10% of cases) [1,2]. Microvascular decompression (MVD) is a curative surgical treatment for medically intractable TN caused by NVC [3] and it makes logical sense. MVD for venous compression alone does not necessarily show significantly inferior effectiveness in relieving pain over arterial compression, although it does show a higher recurrence rate [1,2].

Accurate preoperative diagnosis of NVC is crucial for deciding whether to perform MVD. Advanced high-spatial-resolution MRI is associated with a high diagnostic certainty for NVC and is a reliable tool for its preoperative diagnosis [4-7]. However, conventional MRI has certain disadvantages in delineating venous NVCs in the prepontine cistern compared to arterial NVCs. In the absence of clear visualization of NVC on imaging, patients with TN may miss out on the chance to obtain relief from horrible lancinating facial pain. Improved visualization can be achieved through 3D computer graphics modeling reconstructions with the fusion of multimodal images, as such techniques enable an accurate evaluation of offending vessels in cases where conventional MR images do not clearly show the presence of NVC [8]. We present a case of a patient with TN caused by intraneural venous compression that was challenging to visualize. This report highlights the extent to which the precise anatomical features can be visualized with modern imaging technology before performing MVD.

## Case presentation

Imaging data acquisition and processing

MRI data were acquired with a 3.0-Tesla MRI system (MAGNETOM Verio, Siemens, Munich, Germany). The scanning protocol included a 3D constructive interference in steady state (CISS) sequence (TR, 6.31 ms; TE, 2.68 ms; flip angle, 40°; field of view, 150x150 mm; matrix, 320x288; slice thickness, 0.5 mm) and a 3D time-of-fight magnetic resonance angiography (TOF-MRA) sequence (TR, 23 ms; TE, 3.75 ms; flip angle, 18°; field of view, 150x150 mm; matrix, 448x247; slice thickness, 0.7 mm).

CT was performed using a 320-slice multidetector CT system (Aquilion ONE, Canon, Tokyo Japan) (slice thickness, 0.5 mm; scan speed, 0.28 s/cycle). For the acquisition of the CT arteriography and venography (CTA/CTV), an iodized contrast medium was administered by intravenous injection, and separate scans were performed to obtain arterial and venous phase data.

The 3D TOF-MRA, 3D CISS, and CTA/CTV were fused and processed using the 3D visualization software GRID® (Kompath, Tokyo, Japan). Using a multi-thresholding method, a vessel model was segmented from the 3D TOF-MRA and CTA/CTV images, and nerve models were segmented from the CISS images. A brainstem and cerebellum model was automatically segmented with a deep learning algorithm. A trigeminal nerve model was segmented with a single-thresholding method. All models were visualized using a surface rendering method.

Case description

**Figure 1:**
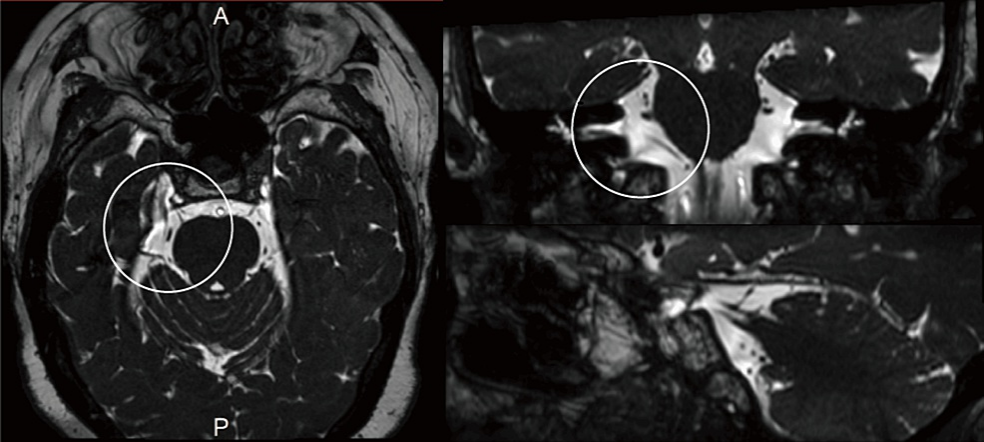
MRI 3D CISS image The image shows no vascular compression anywhere along the trigeminal nerve CISS: constructive interference in steady state; MRI: magnetic resonance imaging

**Figure 2:**
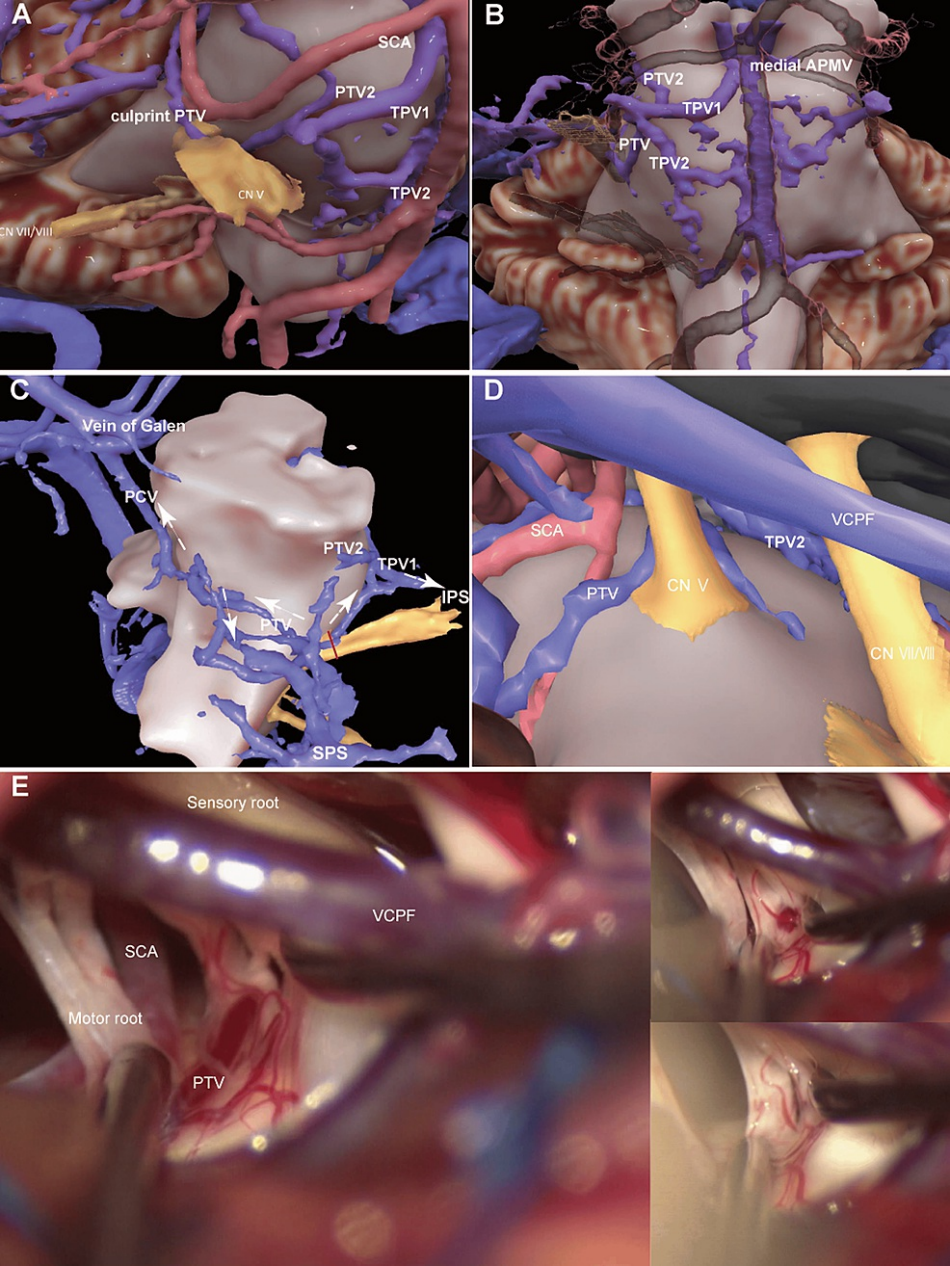
Preoperative 3D computer graphics model and intraoperative view Lateral (A), ventral (B), and dorsal (C) views from the brainstem and simulated operative view (D) of the 3D computer graphics model created by fusing CTV and 3D CISS. The pontotrigeminal vein (PTV) penetrating the sensory root of the trigeminal nerve compresses the trigeminal root entry zone (REZ). The offending vein joins the precentral cerebellar vein (PCV), the superior hemispheric vein, and the transverse pontine vein (TPV), draining to the vein of Galen, superior petrosal sinus (SPS), and inferior petrosal sinus (IPS). In the case of cutting the PTV at the posterior part of the trigeminal nerve (red line), outflow to three directions (white arrow) could be expected (C). Intraoperative view (E) showed findings consistent with the simulated view (D). It was not possible to track where to drain the culprit vein from in the operative view, but using the previous imaging information, the vein was coagulated, cut, and detached completely, paying careful attention to avoid heat injury and extensive manipulation. APMV: anterior pontomesencephalic vein; CISS: constructive interference in steady state; SCA: superior cerebellar artery; VCPF: vein of the cerebellopontine fissure

The patient was a 71-year-old female with a seven-year history of typical TN on the right side of her face. The paroxysmal and lancinating pain was distributed in the V3 territory, and the patient had difficulty eating as chewing triggered the pain. Treatment with carbamazepine was initially effective but gradually became less effective and the patient was referred to another hospital. However, a surgical option was not indicated since a clear neurovascular conflict was not detected on MRI. The patient eventually developed a side effect of drowsiness due to the medication and consulted our hospital seeking alternative treatment. MRI examination at our hospital revealed no vascular compression around the trigeminal nerve, as in the previous radiological findings (Figure 1). However, a surgical simulation with a 3D computer graphics model revealed compression of the trigeminal REZ with the pontotrigeminal vein (PTV) passing through the nerve (Figure 2).

The patient underwent MVD via the retrosigmoid approach. Intraoperatively, we confirmed that the PTV penetrated the nerve, and no other offending vessels were detected. The culprit PTV was deemed impossible to transpose and was therefore coagulated, cut at some point distant from the nerve to avoid heat injury, and gently pushed out of the nerve (Figure 2). This procedure was made possible because the 3D computer graphics model showed the presence of the other collateral venous outflow, meaning that if the penetrating PTV was transected, the blood flow on the rostral side could be expected to divert to the vein of Galen via the precentral cerebellar vein, or to the superior petrosal sinus (SPS) via the cerebellar vein coursing on the hemispheric cerebellar surface, and the blood flow on the caudal side would drain into the transverse pontine vein (TPV), which would likely drain directly into the superior part of the inferior petrosal sinus. The patient did not experience any postoperative complications, and her pain improved immediately after the operation, with complete pain relief being delayed but achieved within a month. The patient remains pain-free more than two years later.

## Discussion

Although venous compression is a known cause of TN, it remains a topic of debate. Several reports on the surgical outcomes of purely venous compression have been published, providing mounting evidence about the difficulty of surgical management compared with arterial compression [1,2]. It is difficult to be certain that venous compression is present preoperatively, and hence an accurate preoperative diagnosis of the venous type is desirable. MRI is certainly the gold standard for the preoperative detection of NVC before MVD. With the advancements in MRI technology, several potentially suitable sequence combinations have been explored. Examinations are generally based on high-spatial-resolution heavily T2-weighted imaging such as 3D balanced steady state gradient echo (CISS, FIESTA, bFFE) and 3D fast turbo spin echo (SPACE, DRIVE) sequences that allow for fine visualization of the vascular-nervous structure in the cerebellopontine angle, along with 3D time-of-flight magnetic resonance angiography for the differentiation of arterial and venous vasculature and high-resolution gadolinium-enhanced T1-weighted imaging for the detection of smaller veins. The higher signal obtainable at 3.0 Tesla enables better visualization than lower magnetic field scanners.

Although these MRI sequences and combinations demonstrate highly accurate detection of NVCs [4-7], venous compression is still often identified intraoperatively in patients with false-negative MRI findings [7,8]. Recognition of venous NVC may sometimes require neurosurgical experience, and conventional 2D MRI has limited ability to visualize veins with low flow-related signals and the relationships between nerves and blood vessels when they run parallel and in close contact. Thus, the advent of image fusion and 3D model reconstruction is expected to aid in overcoming these difficulties. Such methods for overcoming the limitations of MRI sequences can provide an overview of the spatial relationships of nerves and vessels, which can be observed from different perspectives, enabling a more accurate preoperative diagnosis of NVC [9-13], and ultimately improving detection of venous NVCs [8]. In this case, we used multimodal image fusion of MRI and multi-detector CT images with high spatial resolution, as previously described [14]. This combination not only took advantage of clear bone anatomy derived from CT but also allowed the delineation of thinner vessels, particularly veins, than the original MRI.

The difficulty in the surgical management of venous-type TN can be attributed to the high level of individual anatomical variations in the venous system, the fragile nature of the veins, and adherence to adjacent nerves. There are some cases where the neurosurgeon is forced to sacrifice culprit veins, sometimes resulting in serious complications stemming from drainage obstruction. Many investigators suggest that smaller veins or minor tributaries with identifiable collateral drainage can be safely sacrificed after attempting to preserve the veins by transposition or interposition [2,15,16]. Although it is of practical importance to precisely evaluate the size, anastomosis, and drainage pattern of veins on preoperative imaging, there is currently no reliable preoperative method for evaluating collateral flow for the safety of venous sacrifice. Moreover, while MVD is an elegant keyhole microsurgical technique, the surgical corridor is restricted. It is difficult to identify and track the offending vein, even with endoscopy, especially when a deep vein of the transverse type, such as the TPV or PTV, does not drain into the superficial superior petrosal venous system, which is encountered while approaching the trigeminal nerve.

The safety of sacrificing offending TPVs with respect to leaving sufficient collateral outflow has been described [14,16,17]. PTVs located on the posterior mesencephalic surface mostly communicate with a few veins emptying into the Galenic draining group superiorly, and with the petrosal draining group inferiorly [18]. However, such findings do not blindly endorse the sacrifice of these veins. In the present case, the culprit PTV was deeply located and it was difficult to identify the drainage point in the operative field, which posed a challenge for visualizing the offending vein preoperatively and its surgical management. However, instead of merely recognizing that the vein may be in contact with the trigeminal nerve and may be sacrificed, we explicitly realized that the vein penetrated the trigeminal nerve and was able to be sacrificed without resultant venous complications based on the additional information about the intricate venous network. Although the question as to why a congenitally penetrating vein can be attributed to the expression of symptoms remains unanswered, in intraneural venous-type TN, the sacrifice and complete detachment of intraneural veins is considered the best surgical choice if possible, partly because the extensive manipulation of the nerve may lead to uncomfortable sensory deficits [19,20].

## Conclusions

Surgical simulation utilizing 3D computer graphics imaging proved to be an effective tool in managing a patient with TN in whom the presence of NVC was uncertain on conventional MRI, helping achieve good surgical outcomes. A comprehensive preoperative knowledge of anatomical features is crucial for identifying patients who can benefit from MVD and helps avoid unfavorable surgical outcomes.
